# Pericardial Effusion: A Novel Presentation of Aplastic Anemia

**DOI:** 10.7759/cureus.33276

**Published:** 2023-01-02

**Authors:** Robert J Hall, Daniel F Leach, Ellery Altshuler, Robert P Seifert, Zeina A Al-Mansour

**Affiliations:** 1 Department of General Internal Medicine, University of Florida Health, Gainesville, USA; 2 Internal Medicine, Southeast Health Medical Center, Dothan, USA; 3 Department of Radiation Oncology, University of Virginia Health, Charlottesville, USA; 4 Department of Radiation Oncology, University of Florida Health, Gainesville, USA; 5 Department of Pathology, Immunology, and Laboratory Medicine, University of Florida Health, Gainesville, USA; 6 Department of Hematology and Oncology, University of Florida Health, Gainesville, USA

**Keywords:** alkylating/hypomethylating agents, immunosuppressive therapy, myelodysplastic syndrome, aplastic anemia, pericardial effusion

## Abstract

Pericardial effusion is defined as the accumulation of fluid between the visceral and parietal pericardium. The underlying etiology varies as any pathology that causes pericarditis or involves the pericardium can cause effusion. In practice, the majority of pericarditis cases are idiopathic, although these are assumed to be secondary to occult viral infection or inflammatory phenomena. Malignancy, particularly the metastatic spread of noncardiac primary tumors, has been implicated as a differential in the diagnosis of pericardial effusion. Though commonly seen in solid malignancies, effusion has been reported in hematologic malignancies such as myelodysplastic syndrome (MDS), acute leukemia, and lymphoma.

Nonetheless, pericardial effusions associated with hematologic conditions are extremely rare with only one case report published describing pericardial effusion secondary to immune thrombocytopenia (ITP). We herein report the first documented case, to our knowledge, of pericardial effusion as an initial clinical manifestation of aplastic anemia in a middle-aged male presenting with pancytopenia.

## Introduction

Pericardial effusion associated with a cancer diagnosis is not uncommon. Pericardial effusion present with myeloproliferative disorders and other malignancies is most often due to the direct extension of neoplasm into the pericardium. However, with myelodysplastic syndrome (MDS), pericardial effusion appears to be immune-mediated [1,2] or treatment-related [3,4]. Additionally, patients with MDS are immunocompromised due to bone marrow suppression, which can lead to infectious pericardial effusions [5]. The chemotherapeutics of various classes (i.e., azacitidine, cytarabine, fludarabine, doxorubicin, docetaxel, cyclophosphamide, and dasatinib), disease-modifying immunotherapies (i.e., alemtuzumab and immune checkpoint inhibitors), and radiation therapy can cause pericardial disease with effusion [6]. Additionally, autoimmune phenomena associated with malignancy have been postulated to cause an inflammatory effusion in patients without evidence of infection or direct invasion by malignancy [7].

Pericardial effusion has not been reported in the published literature in the setting of aplastic anemia, although it has been reported in the setting of treatments used in aplastic anemia including cyclosporine and alemtuzumab (specifically after hemopoietic stem cell transplant). However, these reports were of patients being treated for diseases other than aplastic anemia [8,9].

## Case presentation

A male in his late 50s with a history of type 2 diabetes mellitus, hypertension, hyperlipidemia, gout, and stage 3A chronic kidney disease presented with a five-month history of progressive shortness of breath, fatigue, and lower extremity edema. A trial of furosemide by his primary care physician did not alleviate his symptoms, after which a complete blood count (CBC) showed marked pancytopenia, specifically moderate macrocytic anemia (hemoglobin of 8.6 g/dl), mild neutropenia (white blood cell count of 3,400/μl), and severe thrombocytopenia (platelet count of 1,600/μl). Peripheral smear showed moderate microcytic anemia with few spherocytes, dimorphic red blood cell (RBC) population with few micro-ovalocytes, mild neutropenia with no left shift or morphological abnormalities, and severe thrombocytopenia.

During initial hospitalization, CBC showed severe pancytopenia with hemoglobin of 5.2 g/dl, white blood cell count (WBCs) of 1,800/μl, and platelet count of 3,000/μl. Anemia workup included iron panel, reticulocyte count, vitamin B12 and folate levels, and hemolysis laboratory tests (lactate dehydrogenase and haptoglobin). Iron panel showed slightly elevated serum ferritin of 624.4 ng/mL, elevated serum iron of 285 μg/dl, and 100% iron saturation, though this was in the context of a recent RBC transfusion. Corrected reticulocyte count was 0.1%. Vitamin B12 was elevated at 960 pg/mL; folate was not reported. Lactate dehydrogenase and haptoglobin were normal. Workup for thrombocytopenia included coagulation studies, heparin-induced thrombocytopenia antibody, and full infectious disease workup, all of which were nonrevealing. A computed tomography (CT) scan of the chest showed some borderline lymph nodes, and a CT scan of the abdomen/pelvis showed mesenteric lymphadenopathy, but these findings resolved on subsequent imaging.

As such, bone marrow biopsy was performed and demonstrated the absence of platelet-making megakaryocytes with no overt dyspoiesis in granulocytes and erythroid precursors, normal marrow cellularity (1-2:1 fat-to-cell ratio), and normal iron stores. There was no evidence of acute leukemia, lymphoma, plasma cell myeloma, myelofibrosis, or granulomas. Karyotype and fluorescence in situ hybridization did not show any abnormalities. Flow cytometry was supportive, showing no overt immunophenotypic evidence of non-Hodgkin’s B-cell lymphoproliferative disorder, apparent T-cells, or acute leukemia. Additionally, no paroxysmal nocturnal hemoglobinuria clone was identified. Next-generation sequencing (NGS) showed no evidence of mutations, but lack thereof can be seen in aplastic anemia, chronic myelogenous leukemia, and chronic lymphocytic leukemia. These findings, specifically the amegakaryocytic thrombocytopenia in conjunction with normal cellularity, did not fully explain the blood counts, which raised the question of occult chronic immune thrombocytopenia (ITP) versus MDS. Due to the severity of the patient’s symptoms and pancytopenia, a biopsy was repeated to rule out MDS but showed similar results. The patient did not respond to a trial of high-dose intravenous immunoglobulin (IVIG) and dexamethasone, so weekly rituximab was started at 375 mg/m^2^. At this time, the patient was noted to have a large pericardial effusion, which was drained. Pericardiocentesis performed during initial hospitalization yielded 1,500 mL of fluid. Bacterial, fungal, and acid-fast bacilli stains and culture were negative. The microscopic examination of fluid revealed atypical cells favoring reactive mesothelial cells with background chronic inflammation. The fluid was not sent for cytology.

**Figure 1:**
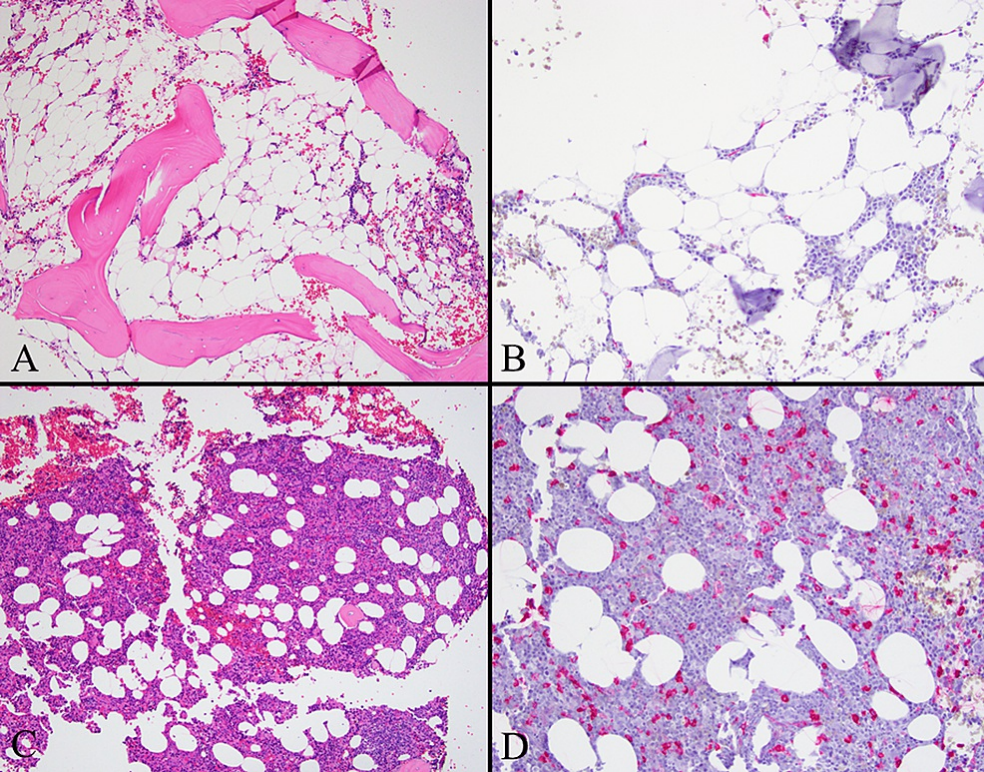
Transformation of SAA to MDS. (A) H&E 100× of bone marrow biopsy at the time of initial aplastic anemia diagnosis. Only scant marrow elements (5% cellularity) are present with no increase (1%) in CD34(+) blasts. (B) CD34 immunohistochemistry 200×. (C) H&E 100× of bone marrow biopsy 11 months later, showing a hypercellular bone marrow (70%-80% cellularity), erythroid hyperplasia, and increased (10%-12%) CD34(+) blasts. (D) CD34 immunohistochemistry 200×. SAA, severe aplastic anemia; MDS, myelodysplastic syndrome; CD34, cluster of differentiation 34.

Unfortunately, his pancytopenia progressed with a hemoglobin of 5.2 g/dl from 9.6 g/dl, leukopenia of 1,800/µl from 5,100/µl, and rapid severe thrombocytopenia; one CBC showed a platelet count of 0/µl from 3,000/µl. At that point, he was transferred to a tertiary care center for further workup. A subsequent third biopsy performed 4-5 months after the initial biopsies showed 5% cellularity with decreased trilineage hematopoiesis (Figure 1).

No overt dyspoietic changes were appreciated, and cytogenetic and molecular testing were unremarkable. Thus, the findings were felt to be most consistent with severe aplastic anemia (SAA).

**Figure 2:**
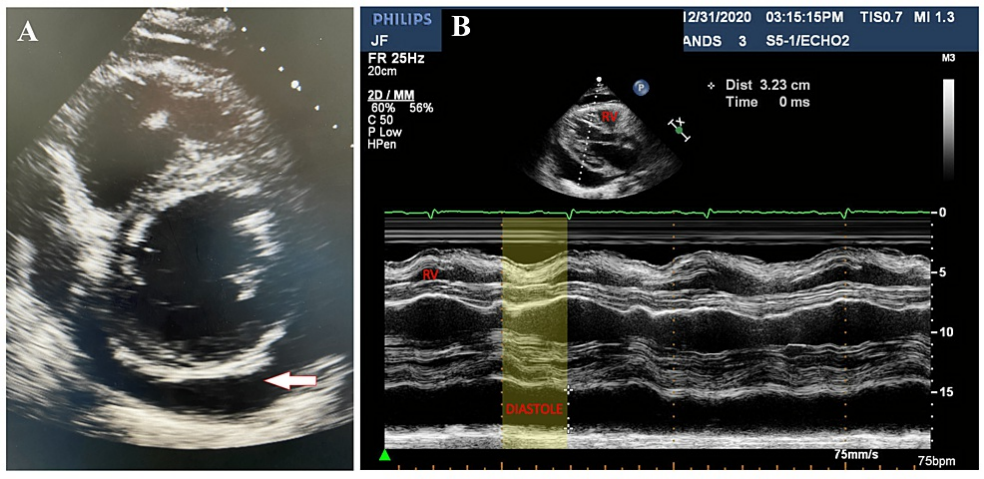
Echocardiography of pericardial effusion. (A) Parasternal short axis view of echocardiogram at the basal level of the left ventricle demonstrating an anterior/posterior view of the pericardial effusion (indicated by white arrow). (B) M-mode demonstrating diastolic collapse of the right ventricle (indicated by yellow shade) secondary to the pericardial effusion.

Interestingly, a small, asymptomatic pericardial effusion had reaccumulated one month after his discharge. Over the subsequent months, the patient remained asymptomatic, and repeated echocardiography did not show signs of hemodynamic compromise, valvular dysfunction, or tamponade. During readmission, the patient remained hemodynamically stable. However, echocardiography 6.5 months after initial presentation showed an enlarged, basal, posterior effusion with signs of hemodynamic compromise, including right atrial systolic collapse, right ventricular diastolic collapse, and abnormal respiratory variation in early diastolic mitral and tricuspid inflow (Figure 2).

With the recurrence of the pericardial effusion, the decision was made to proceed with a subxiphoid pericardial window due to the posterior placement of the effusion. Sanguineous fluid (900 mL) was removed at the time of the operation. Cytology was negative for malignancy. Pericardial biopsy showed fibrotic pericardial tissue without significant inflammation and negative fungal Grocott’s methenamine silver (GMS) stain. Fluid analysis was not conducted. Post-procedure echocardiography performed two months later showed a smaller pericardial effusion without signs of valvular dysfunction or hemodynamic compromise.

Due to pancytopenia secondary to SAA and the presence of bleeding hemorrhoids on readmission, the patient received frequent platelet transfusions and one packed red blood cell transfusion to maintain his platelets above 20,000/μl and hematocrit above 20%. He developed hives after platelet transfusion, requiring diphenhydramine treatment and premedication. Regarding immunosuppressive therapy for SAA, horse antithymocyte globulin (hATG) was administered for three days followed by cyclosporine 175 mg twice daily with target levels of 200-400 ng/mL for six months. Eltrombopag 75 mg once daily was started for six months to stimulate platelet production.

Unfortunately, the patient became transfusion-dependent, and bone marrow transplantation (BMT) was persistently delayed by infectious complications. The treatment regimen of hATG/cyclosporine/eltrombopag was complicated by *Clostridium difficile* colitis. He was also noted to have a perirectal wound likely to be an early decubitus ulcer as CT scan of the abdomen/pelvis did not show underlying abscess. Blood cultures exhibited no growth; *C. difficile*, human herpes virus-6 (HHV-6), and gastrointestinal (GI) polymerase chain reactions (PCRs) were negative; and *Aspergillus* galactomannan was negative. As surgical intervention for the perirectal wound was not deemed necessary, the patient was treated empirically with levofloxacin and metronidazole. During pretransplant evaluation, the patient was found to have monosomy 7 MDS by bone marrow biopsy rendering him ineligible for BMT. He began treatment with decitabine as repeat biopsy showed high-grade MDS; venetoclax was held due to intolerance and neutropenic fever. Peripheral flow cytometry demonstrated 5% blasts, which, in conjunction with worsening cytopenias, was concerning for disease progression. This was supported by the development of recurrent, bilateral, sanguineous pleural effusions, which initially showed reactive mesothelial cells but subsequently exhibited abnormal myeloblasts with aberrant expression of cluster of differentiation 7 (CD7) and cluster of differentiation 56 (CD56) by flow cytometry, concerning for conversion of MDS to acute leukemia. The fluid analysis of the pleural effusion was supportive, showing an increase in WBC count from 70 to 300 and a decrease in the RBC count from 82,000 to 3,400.

Ultimately, the patient died from complications from MDS. He presented to the BMT clinic “not feeling well” with nausea and dry heaving but normal vital signs. He was given intravenous normal saline, magnesium, and ondansetron in the outpatient setting and discharged home in stable condition. He died three days later prior to follow-up. Of note, he was profoundly immunosuppressed and had pancytopenia immediately prior to his death with WBCs of 1,700/μl, absolute neutrophil count (ANC) of 650/μl, hemoglobin of 7.3 g/dl, and platelet count of 78,000/μl.

## Discussion

The etiology of pericardial disease with effusion can be broadly categorized as infectious or non-infectious, with non-infectious causes being further subclassified as neoplastic, traumatic, metabolic, immunologic, and idiopathic [7,10]. A wide variety of organisms can infect the pericardium, though infection resulting in purulent effusion is more frequently associated with bacterial and fungal infections [5]. While no specific cause is found in the great majority of cases of pericarditis, a specific cause is more often found in cases of large pericardial effusions [11].

Aplastic anemia can be immunocompromising when WBC counts are dangerously low. However, our patient had an absolute neutrophil count of 2,710/μl on initial presentation, which is sufficiently high to decrease the likelihood of opportunistic bacterial and fungal infections. Furthermore, the analysis of pericardial fluid was not consistent with bacterial or fungal infection. Viral infections are often presumed to be the source of pericardial effusions that are not otherwise found to have a specific cause. They cannot be ruled out in our patient as pericardial fluid was not sent for PCR. However, viruses are much less likely to cause large effusions, as in our case, and effusions without concomitant pericarditis [11].

The patient had a history of two metabolic processes, namely, chronic kidney disease and gout, both of which can be associated with pericarditis and pericardial effusions. However, the patient was not acutely uremic nor in a gout flare upon presentation. Thus, these causes were considered unlikely. Most effusions caused by a neoplastic process are the result of direct extension of tumor into the pericardial space, and our patient had no evidence of such a neoplastic process as the initial pericardial fluid did not show malignant cells but rather reactive mesothelial cells, although formal cytology was not performed at that time. Pericarditis with hemopericardium secondary to severe thrombocytopenia was also considered, but the initial pericardial fluid was not sanguineous. Furthermore, pericardial biopsy during the subxiphoid pericardial window procedure showed fibrotic pericardial tissue without significant inflammation, lowering acute pericarditis on the differential. Sarcoidosis can present with pericardial effusion and aplastic anemia [12], but subsequent imaging did not reveal hilar lymphadenopathy, and bone marrow biopsy did not show granulomas.

Aplastic anemia is caused by an insult to hematopoietic progenitor cells, which can be autoimmune in nature, and it is possible that such an autoimmune process could simultaneously cause inflammation and effusion of the pericardium. While cyclosporine used to treat aplastic anemia has been implicated in pericardial effusions, our patient was treatment-naïve when he presented with an effusion. Inherited bone marrow failures, predisposition syndromes, and GATA2 deficiency, which can present with thrombocytopenia and lymphedema [13], were ruled out by NGS, which showed no mutations.

To our knowledge, this is the first case report of aplastic anemia to present primarily as pericardial effusion. Several cases were previously reported describing pericardial effusions secondary to cytotoxic agents and disease-modifying therapies in the treatment of various neoplastic and nonneoplastic conditions. One report discusses alemtuzumab [9]. Scattered reports, as well as the European Society of Cardiology guidelines, discuss cyclosporine-associated [8,14] and sirolimus-associated pericardial effusions [15]. First-line treatment for SAA is triple immunosuppression with hATG, cyclosporine, and the thrombomimetic agent eltrombopag (rarely romiplostim) [16]. Except for cyclosporine, none of these medications used for aplastic anemia have ever been reported in association with pericardial effusion or pericarditis in the literature. Of note, cyclophosphamide is a salvage therapy for SAA but causes hemorrhagic pericarditis in high doses. Cyclophosphamide may cause effusion and cardiomyopathy by an unclear mechanism. The current theory is that cyclophosphamide metabolites cause oxidative stress and direct endothelial capillary damage [17].

Aplastic anemia may coexist with or evolve into another hematologic disorder, particularly paroxysmal nocturnal hemoglobinuria, MDS, or acute myeloid leukemia. Interestingly, few cases of pericardial effusion have been reported in association with MDS [1-4], as well as case reports describing viral [18] or autoimmune [19,20] pericardial effusions in the setting of allogeneic hematopoietic cell transplant for MDS as well. An autoimmune mechanism has been proposed to explain an intrinsic link between pericardial effusion and MDS [1,2]. In one case, a 34-year-old female with MDS developed erythema nodosum and pericardial effusion, which improved with corticosteroid taper, suggesting immune-mediated pathophysiology. Of note, the taper improved her thrombocytopenia highlighting, at least partially, the potential immune-mediated mechanism of cytopenia in MDS. Several reports describe abnormal T-cell responses to antigen presentation, as well as abnormal B/T-cell communication in MDS, which may lead to maladaptive immune responses [1]. Further literature illustrates that therapy with alemtuzumab, azacitidine, cyclophosphamide, or fludarabine in the treatment of MDS has been associated with pericardial effusion [3,4,14].

In our case, the pericardial effusion was also likely immune-mediated as indicated by the pericardiocentesis findings. Infectious etiology was ruled out since the patient never developed fever and had negative microbial culture and serological data. Neoplastic etiology was also ruled out as flow cytometry and cytology were negative for blasts in the pericardial fluid. Furthermore, since the onset of pericardial effusion preceded the initiation of hATG, cyclosporine, and eltrombopag, none of these agents could be the culprit for our patient’s recurrent effusions. Up to 15% of aplastic anemia cases transform to MDS. The patient in our case eventually progressed to MDS and was treated with decitabine and venetoclax. While decitabine is a hypomethylating agent similar to azacitidine, it has never been reported in association to pericardial effusion, whereas azacitidine has in some case reports [3,4]. The fact that his aplastic anemia evolved into MDS in a relatively short period of time (18 months) further supports the theory that his pericardial effusion was immune-mediated and strongly linked to evolving MDS in the background of SAA.

## Conclusions

Pericardial effusions are characterized by the accumulation of greater than 50 mL of fluid between the visceral and parietal pericardium. They are a commonly encountered finding in clinical practice but not always associated with concomitant pericarditis. The differential diagnosis is broad but can be simply classified as infectious or non-infectious with non-infectious causes being further subclassified as neoplastic, traumatic, metabolic, immunologic, or idiopathic. Despite this broad differential, in most cases of pericardial effusion, particularly small pericardial effusions, no etiology is found.

In this report, we describe a case of aplastic anemia that presented with a large inflammatory pericardial effusion in the absence of pericarditis and posit a shared autoimmune phenomenon as a potential explanation for the finding. Furthermore, several therapies used in hematologic malignancies can cause pericardial effusions, but two to consider are as follows: (1) immunosuppressive drugs used for SAA or in the setting of hematopoietic stem cell transplant (i.e., cyclosporine, sirolimus, and alemtuzumab) and (2) alkylating/hypomethylating cytotoxic agents used for myeloid malignancies (i.e., azacytidine, cytarabine, cyclophosphamide, and fludarabine).
